# The special role of CXCL13 in Lyme neuroborreliosis: a case report

**DOI:** 10.1186/s42466-022-00167-y

**Published:** 2022-01-17

**Authors:** Deborah K. Erhart, Vera Bracknies, Susanne Lutz-Schuhbauer, Sonja Wigand, Hayrettin Tumani

**Affiliations:** 1 Specialty Hospital of Neurology Dietenbronn, Dietenbronn 7, 88466 Schwendi, Germany; 2 Department of Neurology, University of Ulm, Oberer Eselsberg 45, 89081 Ulm, Germany

**Keywords:** Lyme neuroborreliosis, CXCL13, Cerebrospinal fluid, Meningoencephalitis

## Abstract

The diagnosis of chronic lyme neuroborreliosis can be a challenge even for experienced neurologists. The clinical picture may be multifaceted, including polyradiculitis to cranial nerve palsies, meningitis, encephalomyelitis, encephalopathy and peripheral neuropathy. We report on a patient presenting with basal leptomeningoencephalitis associated with vasculopathy where the chemokine CXCL13 in cerebrospinal fluid played an important diagnostic role.

## Case report

A 51-year-old female patient was admitted to our neurological hospital with sharp headache and neck pain persisting for six months. In addition, she described a general feeling of weakness, fatigue, concentration and short-term memory problems for 3–4 weeks. She also noticed dizziness, double vision and balance disorders. She had unintentionally lost 10 kg of weight in the last six months. The family history was positive for multiple sclerosis.

Preliminary examinations included a magnetic resonance imaging (MRI) of the head and cervical spine approximately four months ago. No particular abnormalities were detected from the MRI.

Neurological examination revealed positive Lhermitte's sign, double images standing side by side while looking straight ahead, a gaze direction nystagmus to the right, hyperreflexia of legs and an ataxic gait pattern with signs of sensory ataxia.

**Fig. 1:**
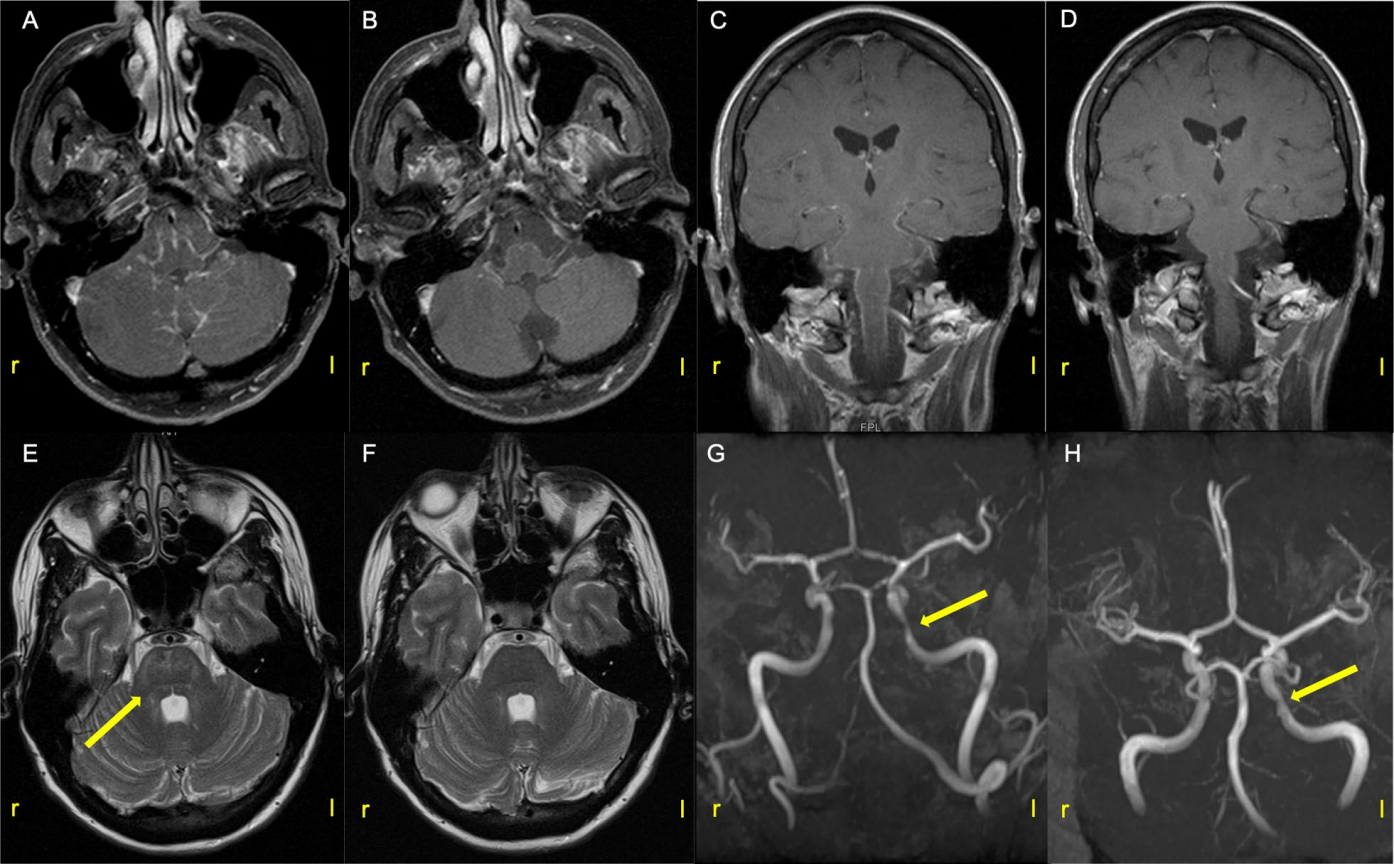
MRI of the brain shows gadolinium-enhancement along the brainstem, cerebellum, occipital and temporal sulci (**A** and **C** before treatment), which decreased during the course (**B** and **D** ten days after). T2-hyperintesities could be detected in the brainstem up to the diencephalon (primarily thalamus and parts of the capsula interna on both sides). **E** shows the MRI before start of treatment, **F** four months after. **G** shows MR-TOF angiography with distal ACI stenosis on the left, **H** 4 months later (arrows). **A**, **B** axial T1; **C**, **D** coronal T1; **E**, **F** axial T2; **G**, **H** MR-TOF angiography

A spinal MRI showed mild degenerative changes without explanation for the gait disorder. Sensory evoked potentials showed demyelination in arms and legs. Cerebrospinal fluid (CSF) analysis revealed an inflammatory process consisting of lymphocytic pleocytosis of 95 cells/µl with activated lymphocytes and plasma cells, elevated lactate (3.6 mmol/l), severe elevation of albumin quotient (44.3 × 10^–3^), and CSF-unique oligoclonal IgG bands (Table 1). We immediately started multifaceted anti-infective therapy with 2000 mg ceftriaxone/day, 750 mg acyclovir/day and 6000 mg/3000 mg ampicillin/sulbactam/day. An MRI of the neurocranium showed gadolinium-enhancement only around pons, medulla oblongata, cerebellum, midbrain, temporal and occipital sulci (Fig. 1A/C). T2-hyperintensities could be detected in the area of brainstem up to diencephalon (primarily thalamus and parts of capsula interna on both sides; Fig. 1E). There were no signals in diffusion-weighted imaging. Regarding potential differential diagnoses of basal leptomeningitis, CSF, serum and/or culture for herpes simplex virus-1, varicella zoster virus, listeria, cryptococcus and mycobacterium were negative. There was also no evidence of neurosarcoidosis, glial fibrillary acidic protein-associated encephalomyelitis or neurosyphilis. Finally, a highly elevated CXCL13 value > 5000 pg/ml (normal range < 20 pg/ml) and a positive Borrelia-burgdorferi-specific-IgG-antibody index of 12 (normal range < 1.4) were obtained. Considering the temporal aspect of the patient's symptoms and the CSF findings on intrathecally produced Borrelia antibodies and severely elevated CXCL13, the diagnosis of chronic neuroborreliosis with inflammatory activity was made. Under therapy with ceftriaxone, CXCL13 dropped fastest in contrast to the other CSF parameters such as cell count and antibody index (Table 1). As MR-TOF (time of flight) angiography revealed a distal stenosis of the left internal carotid artery suggesting a vasculitic process, we extended the antibiotic treatment with cortisone (Fig. 1G). In follow-up examinations (10 days and 4 months later), hardly any more gadolinium-enhancement or T2-hyperintensities of the brain parenchyma could be detected (Fig. 1B/D/F). The stenosis of the internal carotid artery was less pronounced (Fig. 1H). In follow-up four months later, the patient presented with a complete resolution of symptoms except for mild residual fatigue.

**Table 1:** CSF parameters at different time points

Parameter (normal range)	1. LP (day 0)	2. LP (day 7)	3. LP (day 12)
Cell count [/µl] (< 5)	95	177	101
Cytology [%] (L/M/G/P)	62/15/15/7	84/4/-/4	81/10/-/6
Protein [mg/dl] (45)	379	90	123
Lactate [mmol/l] (2.6)	3.6	3.6	2.6
Albumin quotient (L/S, < 7.5)	44.3 × 10^–3^	14.3 × 10^–3^	20.5 × 10^–3^
B.b.-specific AI IgG (< 1.5)	12	13	3.99
B.b.-specific AI IgM (< 1.5)	n.d.	n.d.	n.d.
Oligoclonal bands in CSF (negative)	pos.	pos.	pos.
Oligoclonal bands in serum (negative)	neg.	neg.	neg.
IgG (intrathecal fraction in %)	56	61	21
IgM (intrathecal fraction in %)	76	92	75
IgA (intrathecal fraction in %)	33	72	60
CXCL13 [pg/ml] (< 20)	> 5000	338	96

The table shows the CSF parameters of the basic and special analyses at different time points during the course of the patient’s disease (1: before treatment at diagnostic lumbar puncture (LP), 2: 7 days post-treatment, 3: 12 days post-treatment). L/M/G/P: lymphocytes/monocytes/granulocytes/plasma cells, B.b.: Borrelia burgdorferi, AI: antibody index, n.d.: not detectable, pos.: positive, neg.: negative

## Discussion

The multisystemic disease Lyme borreliosis is one of the most common tick-borne diseases in Europe. Neuroborreliosis occurs in roughly 15% of cases of Lyme disease [1]. The disease is caused by different species of the spirochete bacterium *Borrelia burgdorferi*. The most frequent manifestations of neuroborreliosis include cranial nerve paresis and meningopolyradiculitis [1]. Due to the variety of symptoms and differential diagnoses, as can be seen in our case study, it is often not easy to make a diagnosis. For the diagnosis, an intrathecal borrelia-specific immunoglobulin synthesis and appropriate symptoms (Bell's palsy, nocturnal polyradicular pain) are required in addition to an inflammatory CSF-syndrome [2]. Diagnostic and activity markers such as the B-cell chemoattractant CXCL13 secreted by cells of innate immunity are of enormous value for this [3]. In studies, CSF-CXCL13 together with Borrelia-burgdorferi-specific-antibody index (B.b.-AI) has shown sensitivity and specificity of  > 96% for acute Lyme neuroborreliosis (LNB) [4]. CXCL13 also proved to be a marker of very early neuroborreliosis, as intrathecal B.b.-specific antibody synthesis can still be negative in about 20% of cases [5]. In addition, it was shown to be an important activity marker in studies, with levels falling sharply during therapy [4]. This is in contrast to B.b.-AI, which can persist years after infection [6]. Serological investigations in LNB-patients with regard to CXCL13 did not show elevated levels in contrast to patients with other bacterial diseases of the central nervous system such as neurosyphilis, which is an important differential diagnosis of LNB and is also associated with CXCL13 elevation [7].

Vasculitis in context of neuroborreliosis is a rare complication affecting only 0.3% to 1% of all LNB-patients, so there are only small case series to date [8]. Especially for younger patients with stroke, this should be kept in mind [9].

## Data Availability

The datasets used and/or analysed during the current study are available from the corresponding author on reasonable request.

## Abbreviations

MRI: Magnetic resonance imaging
CSF: Cerebrospinal fluid
TOF: Time of flight
B.b.-AI: Borrelia-burgdorferi-specific-antibody-index
LNB: Lyme neuroborreliosis

## References

[1] Halperin JJ (2015). Nervous system lyme disease. Infectious Disease Clinics of North America.

[2] Rauer S, Kastenbauer S, Hofmann H, Fingerle V, Huppertz H-I, Hunfeld K-P, Krause A, Ruf B, Dersch R, Consensus group (2020). Guidelines for diagnosis and treatment in neurology - Lyme neuroborreliosis. German Medical Science.

[3] Rupprecht TA, Koedel U, Fingerle V, Pfister HW (2008). The pathogenesis of lyme neuroborreliosis: From infection to inflammation. Molecular Medicine.

[4] Senel M, Rupprecht TA, Tumani H, Pfister HW, Ludolph AC, Brettschneider J (2010). The chemokine CXCL13 in acute neuroborreliosis. Journal of Neurology, Neurosurgery and Psychiatry.

[5] Tumani H, Nolker G, Reiber H (1995). Relevance of cerebrospinal fluid variables for early diagnosis of neuroborreliosis. Neurology.

[6] Hammers-Berggren S, Hansen K, Lebech A-M, Karlsson M (1993). Borrelia burgdorferi-specific intrathecal antibody production in neuroborreliosis. Neurology.

[7] Rupprecht TA, Kirschning CJ, Popp B, Kastenbauer S, Fingerle V, Pfister HW, Koedel U (2007). Borrelia garinii induces CXCL13 production in human monocytes through Toll-like receptor 2. Infection and Immunity.

[8] Wittwer B, Pelletier S, Ducrocq X, Maillard L, Mione G, Richard S (2015). Cerebrovascular events in lyme neuroborreliosis. Journal of Stroke and Cerebrovascular Diseases.

[9] Topakian R, Stieglbauer K, Nussbaumer K, Aichner FT (2008). Cerebral vasculitis and stroke in lyme neuroborreliosis. Cerebrovascular Diseases.

